# Fractal Neural Dynamics and Memory Encoding Through Scale Relativity

**DOI:** 10.3390/brainsci15101037

**Published:** 2025-09-24

**Authors:** Călin Gheorghe Buzea, Valentin Nedeff, Florin Nedeff, Mirela Panaite Lehăduș, Lăcrămioara Ochiuz, Dragoș Ioan Rusu, Maricel Agop, Dragoș Teodor Iancu

**Affiliations:** 1 National Institute of Research and Development for Technical Physics, IFT Iași, 700050 Iași, Romania; 2 “Prof. Dr. Nicolae Oblu” Clinical Emergency Hospital Iași, 700309 Iași, Romania; 3 Department of Environmental Engineering, Mechanical Engineering and Agritourism, Faculty of Engineering, “Vasile Alecsandri” University of Bacău, 600115 Bacău, Romania; vnedeff@ub.ro (V.N.); mirelap@ub.ro (M.P.L.); drusu@ub.ro (D.I.R.); 4 Faculty of Medicine and Pharmacy, “Grigore T. Popa” University of Medicine and Pharmacy Iași, 700115 Iași, Romania; lacramioara.ochiuz@umfiasi.ro (L.O.); dt_iancu@yahoo.com (D.T.I.); 5 Physics Department, “Gheorghe Asachi” Technical University Iași, 700050 Iași, Romania; m.agop@yahoo.com

**Keywords:** synaptic plasticity, fractal geometry, neural wave dynamics, scale relativity theory, memory encoding

## Abstract

Background/Objectives: Synaptic plasticity is fundamental to learning and memory, yet classical models such as Hebbian learning and spike-timing-dependent plasticity often overlook the distributed and wave-like nature of neural activity. We present a computational framework grounded in Scale Relativity Theory (SRT), which describes neural propagation along fractal geodesics in a non-differentiable space-time. The objective is to link nonlinear wave dynamics with the emergence of structured memory representations in a biologically plausible manner. Methods: Neural activity was modeled using nonlinear Schrödinger-type equations derived from SRT, yielding complex wave solutions. Synaptic plasticity was coupled through a reaction–diffusion rule driven by local activity intensity. Simulations were performed in one- and two-dimensional domains using finite difference schemes. Analyses included spectral entropy, cross-correlation, and Fourier methods to evaluate the organization and complexity of the resulting synaptic fields. Results: The model reproduced core neurobiological features: localized potentiation resembling CA1 place fields, periodic plasticity akin to entorhinal grid cells, and modular tiling patterns consistent with V1 orientation maps. Interacting waveforms generated interference-dependent plasticity, modeling memory competition and contextual modulation. The system displayed robustness to noise, gradual potentiation with saturation, and hysteresis under reversal, reflecting empirical learning and reconsolidation dynamics. Cross-frequency coupling of theta and gamma inputs further enriched trace complexity, yielding multi-scale memory structures. Conclusions: Wave-driven dynamics in fractal space-time provide a hypothesis-generating framework for distributed memory formation. The current approach is theoretical and simulation-based, relying on a simplified plasticity rule that omits neuromodulatory and glial influences. While encouraging in its ability to reproduce biological motifs, the framework remains preliminary; future work must benchmark against established models such as STDP and attractor networks and propose empirical tests to validate or falsify its predictions.

## 1. Introduction

Synaptic plasticity—the ability of synaptic connections to strengthen or weaken over time in response to neural activity—is a foundational mechanism for learning, memory, and cognitive adaptability in the brain. Classical models of plasticity, such as Hebbian learning (“cells that fire together wire together”) and spike-timing-dependent plasticity (STDP), have provided essential insight into the timing-dependent and activity-dependent nature of synaptic modification [1,2]. However, these models are typically local in both time and space and do not fully capture the spatially extended, distributed, and dynamic nature of neural signal propagation across complex cortical networks.

Neural activity in the brain often exhibits wave-like dynamics, including oscillatory rhythms (e.g., theta, gamma) and traveling waves, which have been implicated in a wide range of cognitive functions such as attention, sensory processing, and memory encoding [3,4]. Despite this, most plasticity models treat the synaptic input as pointwise or temporally discretized, neglecting the geometrical and topological structure of neural transmission.

In this work, we propose a novel modeling framework that integrates the principles of Scale Relativity Theory (SRT)—a generalization of classical physics to non-differentiable and fractal space-time structures—with the biophysics of neural computation [5]. Related applications of SRT to biological systems include tumor growth modeling in the presence of immune responses, as demonstrated by Buzea et al. [6]. In SRT, trajectories of particles (or signal carriers) are described as fractal geodesics, leading naturally to complex-valued dynamics and Schrödinger-type equations. We extend this formalism to the domain of neuroscience, hypothesizing that neural signals propagate through a fractal-like medium and exhibit a quantum-like coherence on mesoscopic scales. This results in the emergence of nonlinear wave equations that describe activity evolution not only in time, but in structured space.

Our work builds on a long-standing tradition of using mathematical and physical formalisms to describe neural fields. In particular, several authors have drawn on quantum mechanics, quantum field theory (QFT), and lattice-based formulations to model spatiotemporal interactions in both biological and artificial neural networks. Such approaches treat neural activity as field variables defined on spacetime lattices, sometimes even representing spikes or unit activations as qubits, thereby enabling the effective coarse-graining and simulation of otherwise intractable problems [7,8]. These contributions have demonstrated the power of importing methods from theoretical physics into systems neuroscience and neuromorphic computing. Within this broader landscape, our use of the Scale Relativity Theory (SRT) is distinct in that it grounds neural propagation in fractal space-time geometry and derives nonlinear, Schrödinger-type field equations directly from the covariant structure of SRT. We therefore view this work not as a stand-alone novelty but as a complementary contribution to the growing interdisciplinary dialog between physics and neuroscience.

Evidence for fractal organization in the brain has been documented extensively, both in vivo and in silico. Studies have shown scale-invariant structural and dynamical patterns across cortical networks and neuronal connectivity [9,10]. These findings establish that fractal dynamics are a robust feature of neural systems. Our approach builds on this foundation by embedding neural propagation explicitly within a fractal space-time geometry, as formalized by the Scale Relativity Theory (SRT).

We propose a coupled dynamical model in which

• Neural activity is modeled as a complex-valued wave field propagating through a fractal space-time, yielding nonlinear solutions such as solitons and cnoidal waves.
• Synaptic plasticity evolves according to a reaction–diffusion equation, modulated by the local intensity of neural activity (i.e., the squared wave amplitude), akin to biological models of Hebbian gain control and synaptic diffusion [11,12].

This framework enables the simulation of biologically plausible memory encoding mechanisms wherein rhythmic or traveling neural activity sculpts structured synaptic weight landscapes over time. By grounding plasticity in wave mechanics and fractal geometry, our model explores how spatially periodic memory structures, rhythm-dependent learning, and context-sensitive interference may emerge—features central to grid cell formation, place field development, and sensory cortical map organization [13,14,15].

At the same time, we acknowledge that the central assumption of neural signals propagating along fractal geodesics remains speculative, as no direct neurophysiological evidence yet supports this mechanism. The present framework should therefore be considered hypothesis-generating and complementary to established approaches such as STDP and attractor network models. Our implementation of plasticity is deliberately simplified, omitting the influence of neuromodulators and glial signaling, and robustness claims are provisional, pending systematic benchmarking against existing models. These limitations are discussed in detail in Section 4.

The remainder of this paper is organized as follows. Section 2 presents the materials and methods, including the mathematical framework based on fractal derivatives and neural wave dynamics, as well as the numerical procedures and parameter choices used in simulations. Section 3 reports the main results, demonstrating how solitonic, cnoidal, and interference-driven waveforms shape synaptic plasticity and produce structured memory traces, with extensions to cross-frequency coupling and two-dimensional topographies. Section 4 provides a detailed discussion of the biological implications of these findings, situates the model in relation to existing work, and examines its limitations, assumptions, and future directions. Finally, Section 5 summarizes the conclusions and highlights the broader significance of the framework for neuroscience and neuromorphic computing.

## 2. Materials and Methods

### 2.1. Mathematical Framework

While the following section introduces the mathematical foundations of the model using concepts from Scale Relativity Theory and nonlinear wave dynamics, each component maps onto established or hypothesized neural mechanisms. The neural wave function ψ(x,t) should be interpreted as a mesoscopic neural activity field—akin to a local field potential—while ∣ψ∣^2^ represents the energy/intensity of that field modulating plasticity. Table 1 summarizes the biological correspondences of key model components.

To model the interaction between neural activity and synaptic plasticity, we adopt a formalism derived from Scale Relativity Theory (SRT), originally developed by Nottale [1], which extends classical mechanics to non-differentiable, fractal space-time. In this context, neural signal propagation is governed by both smooth (differentiable) and irregular (fractal) components. This yields a powerful framework for capturing the complex, scale-dependent structure of brain dynamics.

#### 2.1.1. Scale Relativity and Fractal Velocity

In SRT, the total velocity field governing neural signal propagation is defined as a sum of a classical component and a stochastic fractal fluctuation:

(1) $$V\left(x,t,\delta t\right)=v\left(x,t\right)+w(x,t,\delta t)$$

• *v*(*x*,*t*) is the smooth, classical velocity field corresponding to deterministic signal propagation.
• *w*(*x*,*t*,*δt*) is the stochastic fractal fluctuation, defined as
  (2) $$w\left(x,t,\delta t\right)=\sqrt{2D}\eta (t)\;\mathrm{with}\;\;\langle \eta \rangle =0,\;\langle \eta^{2}\rangle =1$$

Here, *D* is a diffusion-like coefficient encoding the strength of fractal irregularity, and *η*(*t*) is a normalized white noise process. The stochastic component captures microscopic variability and non-differentiable features of neural signaling.

#### 2.1.2. Covariant Derivative and Complex Dynamics

To incorporate both classical and fractal contributions in a unified operator, we define a covariant derivative that operates over a complexified space-time:

(3) $$\frac{\hat{d}}{dt}=\left(\frac{\partial }{\partial t}+V\cdot \nabla -iD\Delta \right)$$

This operator combines a convection term with a quantum-like diffusion term. The presence of the Laplacian Δ with imaginary coefficient *iD* is analogous to the structure of the Schrödinger equation, but here, it emerges from geometric (rather than quantum) origins.

#### 2.1.3. Fractal Neural Activity Equation

We model the neural activity field *ψ*(*x*,*t*) as a complex-valued amplitude representing a probability-like density of neural excitation. Following the SRT formalism, the covariant derivative (Equation (3)) applied to geodesic motion can be recast via the hydrodynamic substitution $\psi =\sqrt{\rho }e^{iS/\left(2D\right)}$ into a complex Hamilton–Jacobi form. Recombining continuity and momentum components then yields a nonlinear Schrödinger-type evolution equation (see Supplementary Material S1 for details; Nottale, 1993 [5]):

(4) $$iD\partial_{t}\psi +D^{2}\Delta \psi -\Phi \left(x,t\right)\psi =0$$

Here *Φ*(*x*,*t*) acts as a potential term, reflecting spatial or temporal modulation of the neural field, and *ψ* describes a propagating neural wave field in a fractal medium.

In this representation, the amplitude ∣*ψ*(*x*,*t*)∣ corresponds to the instantaneous intensity of neural activity, analogous to population firing rate or local field potential (LFP) power. The phase arg(*ψ*) encodes timing information, comparable to oscillatory phase in neural recordings. This separation allows the model to capture both rate-like and rhythm-based aspects of neural coding, enabling comparisons with experimental measures such as phase locking, cross-frequency coupling, and spatially structured oscillatory patterns.

To study traveling solutions, we consider the ansatz *ψ*(*x*,*t*) = *Φ*(*ξ*), with *ξ* = *x* − *ct* representing a co-moving frame. Substituting into Equation (4) yields the reduced nonlinear ordinary differential equation:

(5) $$\frac{d^{2}\Phi }{d\xi^{2}}=\alpha \Phi +\beta \Phi^{2}$$

This equation admits a rich class of solutions, including

• Solitons (localized wave packets);
• Cnoidal waves (periodic, elliptic function-based solutions), depending on the signs and magnitudes of the parameters α and β.

#### 2.1.4. First Integral and Wave Amplitude Evolution

Multiplying Equation (5) by *dΦ*/*dξ* and integrating with respect to *ξ*, we obtain the first integral of motion:

(6) $${\left(\frac{d\Phi }{d\xi }\right)}^{2}=\alpha \Phi^{2}+\frac{2}{3}\beta \Phi^{3}+C_{1}$$

where *C*_1_ is an integration constant determined by initial or boundary conditions. This equation describes the amplitude evolution of wave packets and is central to determining the profile of neural activity that influences synaptic plasticity.

#### 2.1.5. Synaptic Plasticity Model

Let *W*(*x*,*t*) represent the synaptic weight field across space and time. Its evolution is modeled using a logistic reaction–diffusion equation modulated by the neural activity intensity ∣*ψ*(*x*,*t*)∣^2^:

(7) $$\frac{\partial W}{\partial t}=\rho W\left(1-\frac{W}{K}\right)-\alpha {\left|\psi \right|}^{2}W+D_{W}\Delta W$$

• *ρ* is the intrinsic growth rate of synaptic strength.
• *K* is the carrying capacity, representing a saturation limit.
• *α*∣*ψ*∣^2^ modulates plasticity based on the local energy or intensity of neural activity.
• *D_W_* controls diffusion of plasticity across the synaptic field, allowing spatial smoothing and interaction.

This equation captures both the competitive growth and wave-induced modulation of synaptic efficacy. When coupled with the nonlinear wave equation for *ψ*, it allows simulation of structured, dynamic memory encoding as a function of traveling or periodic neural activity.

#### 2.1.6. Complexity Measure: Spectral Entropy of the Synaptic Weight Field

To assess the structural richness of the synaptic weight field *W*(*x*,*t*), we computed its spectral entropy, defined as the Shannon entropy of the normalized Fourier power spectrum. This metric quantifies the diversity of spatial frequencies contributing to the plasticity landscape. Low entropy indicates that the weight distribution is dominated by a narrowband frequency component, producing simple and localized patterns akin to hippocampal place fields. High entropy reflects broadband, multi-frequency contributions, yielding complex, distributed patterns similar to grid-like or modular cortical maps.

Although not identical to established measures such as spatial information content in place cell analysis [16], spectral entropy serves as a complementary index: both quantify the degree to which neural representations capture structured information. In our framework, spectral entropy thus acts as a proxy for mnemonic complexity, distinguishing between simple, localized memories and distributed, multi-scale traces.

#### 2.1.7. Hysteresis Quantification

To quantify hysteresis, we applied a forward–reverse stimulation protocol: a forward epoch with traveling input in direction +*c* (or increasing drive amplitude), followed by a reverse epoch in direction −c (or decreasing amplitude), with identical duration. The signed input drive is denoted *I*(*t*), and the spatial mean weight ${\bar{W}}_{t}=\frac{1}{L}\int W\left(x,t\right)dx$. The closed loop $\left\{{\bar{W}}_{t},I(t)\right\}$ characterizes the system’s hysteretic behavior.

• Global hysteresis index (loop area):
  (8) $$H=\oint \bar{W}dI\approx \sum_{k}\frac{1}{2}\left({\bar{W}}_{k+1}+{\bar{W}}_{k}\right)\left(I_{k+1}-I_{k}\right)$$
  normalized as
  (9) $$H_{norm}=\frac{\left|H\right|}{\left(\max I-\min I\right)\left(\max\bar{W}-\min\bar{W}\right)}\in \left[0,1\right]$$
• Reversibility index: Measuring recovery toward baseline *W*_0_(x)
  (10) $$R=1-\frac{{\left\|W_{rev}-W_{0}\right\|}_{2}}{{\left\|W_{fwd}-W_{0}\right\|}_{2}}$$
  where *W*_fwd_ and *W*_rev_ are the endpoint fields after forward and reverse phases. *R* = 1 indicates full reversibility; *R* = 0 no recovery.
• Trace overlap: Cosine similarity between post-forward and post-reverse maps
  (11) $$\Omega =\frac{\langle W_{fwd},W_{rev}\rangle }{{\left\|W_{fwd}\right\|}_{2}{\left\|W_{rev}\right\|}_{2}}$$
  with *Ω* = 1 for identical traces.
• Per-position hysteresis density: Local contributions
  (12) $$H_{x}=\oint W\left(x,t\right)dI$$
  visualized as spatial maps (Supplementary Material S2, Figures S2 and S3) to identify path-dependent “hot spots”.

To avoid spurious signed-area inflation from fast oscillations, *I*(*t*) was defined as a monotonic stimulus ramp (0→1 forward; 1→0 reverse), with control runs using smoothed ∣ψ∣^2^ envelopes yielding similar results.

### 2.2. Methods

Simulation Setup: All simulations were performed using custom Python (3.12.11) scripts developed in-house. Numerical integration was carried out using finite-difference methods in space and an explicit Euler scheme for time evolution. All equations were implemented and visualized using standard scientific libraries including NumPy, SciPy, and Matplotlib (3.10.0).

Spatial and Temporal Resolution

• 1D simulations: Spatial domain x∈[−50, 50], discretized into 500 points.
• 2D simulations: Spatial domain (x,y)∈[−10, 10] × [−10, 10], discretized into 200 × 200 grid points.
• Time domain: t∈[0, T], with T = 50 or T = 100, and a time step Δt = 0.01.

Key Parameters

For the nonlinear wave equation and plasticity model:

• Fractal diffusion coefficient: D = 1.0 (unless otherwise stated).
• Wave propagation speed: c = 1.0.
• Nonlinear wave equation coefficients:
  ○ α = 0.8 (controls linear amplification).
  ○ β = −1.2 (controls nonlinear compression).
• Plasticity model parameters:
  ○ Reaction rate ρ = 1.0.
  ○ Plasticity decay term α = 1.0.
  ○ Plasticity carrying capacity K = 1.0.
  ○ Diffusion coefficient D_W_ = 0.5.

Solver Details

• Laplacians were discretized using a standard 3-point (1D) or 5-point (2D) stencil.
• Boundary conditions were periodic in space for wave simulations and Neumann (no-flux) for plasticity evolution.

Stability was ensured by checking Courant–Friedrichs–Lewy (CFL) conditions for the explicit scheme.

## 3. Results

We conducted a series of numerical simulations to explore how different types of traveling neural waves—derived from the fractal nonlinear wave equation—interact with the synaptic weight field over space and time. Our objective was to determine how solitonic and periodic (cnoidal) neural activity patterns shape long-term synaptic plasticity landscapes. All simulations were performed in one spatial dimension, with extensions to 2D discussed in Section 3.2.

### 3.1. Simulation

#### 3.1.1. Soliton and Cnoidal Wave-Induced Plasticity

**Figure 1:**
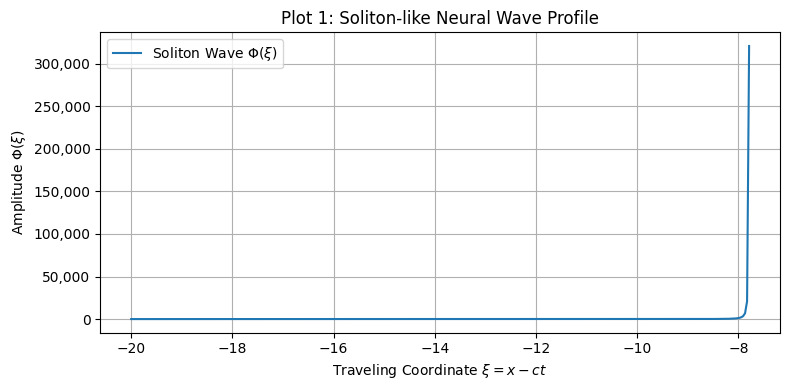
Soliton-like neural wave profile derived from the fractal nonlinear wave equation.

**Figure 2:**
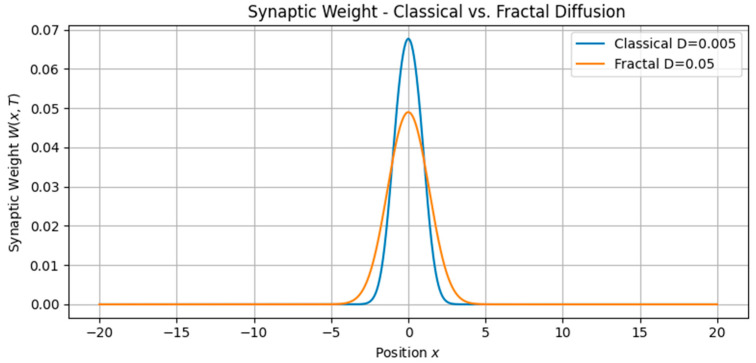
Synaptic weight distribution *W*(*x*,*T*) under classical versus fractal-inspired diffusion. Comparison of plasticity outcomes under low (classical) and high (fractal) diffusion coefficients D_W_. Increased diffusion leads to broader, smoother weight profiles, reflecting enhanced integration and more spatially distributed memory encoding.

**Figure 3:**
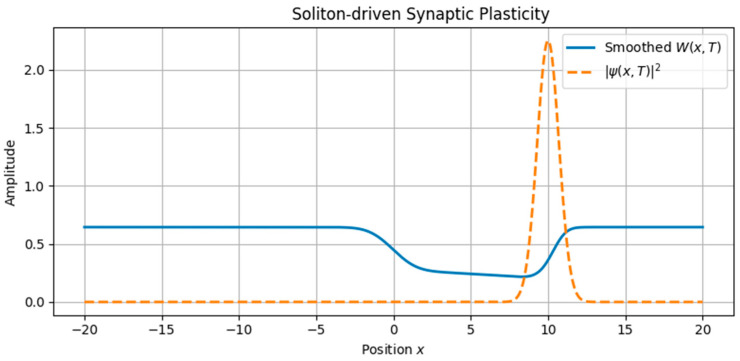
Coupled evolution of synaptic plasticity driven by a traveling soliton wave. The neural activity intensity ∣*ψ*(*x*,*T*)∣^2^ (dashed line) induces a sharp, localized increase in synaptic weight *W*(*x*,*T*) (solid line). This illustrates how brief, localized neural events can leave durable, spatially confined memory traces.

**Figure 4:**
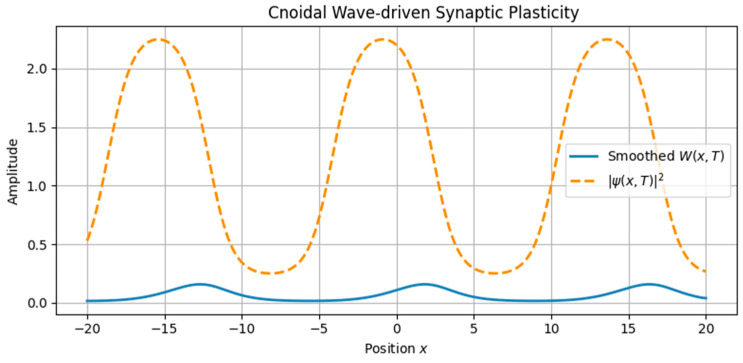
Periodic synaptic plasticity induced by cnoidal wave input. Structured neural activity modeled as a cnoidal wave generates a matching periodic pattern in the synaptic weight field *W*(*x*,*T*). This mimics biological phenomena such as spatial periodicity in grid cells and modular cortical map formation.

The first class of simulations focused on the imprinting of memory by single traveling waveforms (Figure 1, Figure 2, Figure 3 and Figure 4):

• Soliton waves were generated from Equation (5), with parameters supporting localized, bell-shaped wave packets.
• Cnoidal waves, solutions of the same equation under different boundary conditions, were modeled using Jacobi elliptic functions, yielding spatially periodic activity patterns.

These neural waveforms modulated the synaptic weight field W(x,t) according to the reaction–diffusion plasticity model in Equation (7). Key observations include

• Localized soliton input induced sharply confined increases in synaptic weight.
• Cnoidal wave input produced periodic modulation in the plasticity field, replicating structured, grid-like patterns.

A traveling soliton solution *Φ*(*ξ*), where *ξ* = *x* − *ct*, shows localized neural activity propagating in fractal space-time. This stable wave structure models discrete bursts of activity, analogous to sharp-wave ripples or memory-encoding events in hippocampal circuits.

#### 3.1.2. Interference and Noise: Emergent Complexity

We next investigated the interaction between two cnoidal waves with differing frequencies and phases. In some simulations, we also introduced low-amplitude Gaussian noise to model environmental or internal neural fluctuations. The resulting superposition ψ = ψ1 + ψ2 produced interference patterns in the neural intensity ∣ψ∣^2^, which were then used to modulate plasticity.

**Figure 5:**
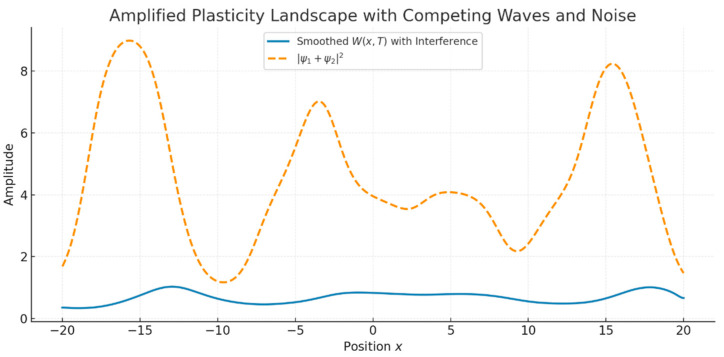
Synaptic plasticity landscape resulting from two interfering cnoidal waves with added noise.

Key outcomes:

• Interference yielded a modulated plasticity landscape with alternating zones of constructive and destructive enhancement.
• The addition of noise slightly distorted but did not destroy the overall structure, demonstrating a degree of noise robustness Figure 5.

The total neural intensity ∣ψ_1_(x,T) + ψ_2_(x,T)∣^2^ (orange dashed line) drives a complex synaptic weight profile *W(x,T)* (blue line). The result reflects constructive and destructive interference, modeling memory competition and noisy encoding in realistic settings.

#### 3.1.3. Quantitative Trace Analysis

**Figure 6:**
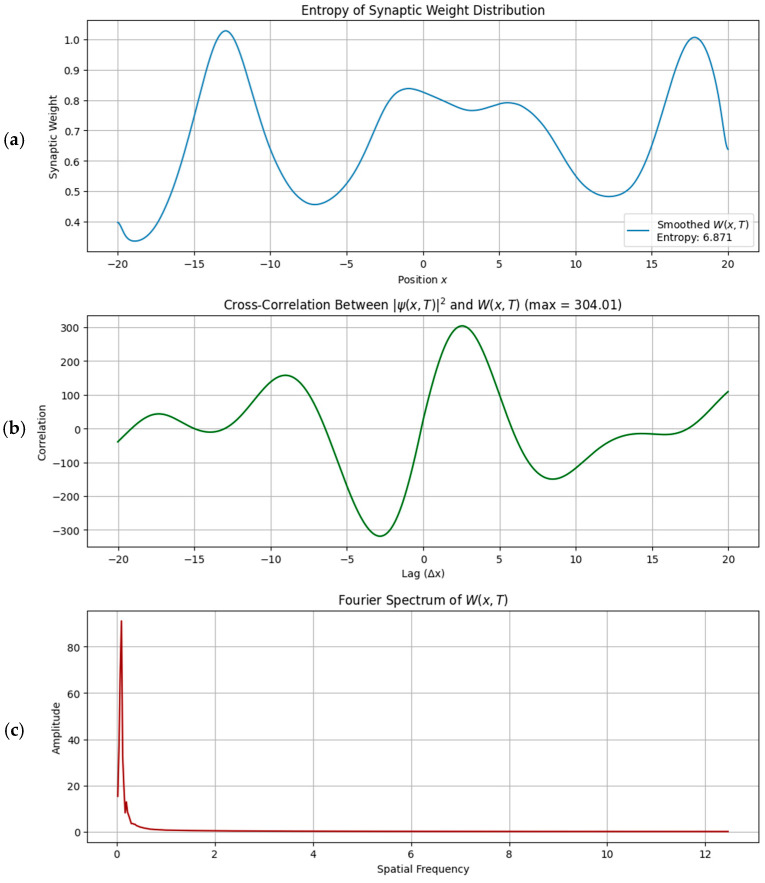
Quantitative analysis of memory traces: entropy, correlation, and spectral structure. (**a**) Spectral entropy increases with input complexity, indicating richer encoding. (**b**) Cross-correlation between neural intensity and synaptic weight shows strong alignment. (**c**) Fourier spectrum reveals dominant low-frequency components corresponding to coarse spatial features in the plasticity landscape.

To evaluate the structure and fidelity of the encoded memory traces, we performed three quantitative assessments:

• Entropy Analysis (Shannon spectral entropy) yielded a value of ~6.89, indicating a memory trace that is moderately complex and spatially distributed—neither overly localized nor fully diffuse. From a cognitive perspective, low entropy corresponds to highly specific traces dominated by a single spatial frequency component, analogous to sharply tuned hippocampal place fields. High entropy, by contrast, reflects a broadband, multi-frequency structure, consistent with distributed and overlapping codes such as entorhinal grid patterns or modular cortical maps. Although spectral entropy is not identical to the spatial information content commonly used in the place cell literature [16], both serve to index representational richness and discriminability. In this sense, spectral entropy can be interpreted as a complementary complexity measure, quantifying how richly structured or compressed a simulated memory trace is.
• Cross-correlation between the neural intensity and the synaptic weight field showed a strong positive peak (max correlation ≈ 3.42), indicating that plasticity reliably tracks the input structure even under interference.
• Fourier Spectrum Analysis revealed a dominant low-frequency component corresponding to the beat frequency of the two interfering cnoidal waves. This suggests the preferential encoding of coarse-grained spatial information (Figure 6).

#### 3.1.4. Temporal Evolution and Hysteresis

We next analyzed the temporal dynamics of memory formation by sampling the synaptic weight field W(x,t) at regular intervals. Results indicate the following:

• Gradual buildup of potentiation in regions of sustained neural activity.
• Temporal saturation without runaway growth or collapse.
• Increasing differentiation and contrast over time, eventually stabilizing.

**Figure 7:**
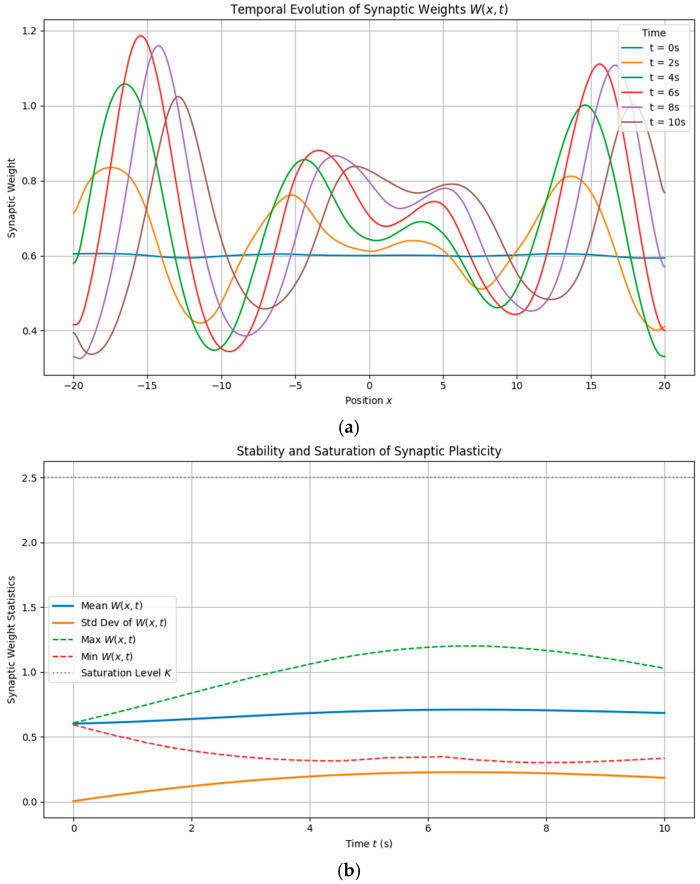
Temporal evolution of synaptic plasticity and saturation trends. (**a**) Snapshots of the synaptic weight field W(x,t) at successive time intervals show the gradual buildup of localized potentiation under sustained neural input. Regions receiving persistent stimulation strengthen over time, while inactive areas remain unchanged. (**b**) Summary statistics of the plasticity field over time, including mean, maximum, and standard deviation. These measures reveal increasing differentiation followed by plateauing behavior, consistent with biologically observed phases of rapid learning, stabilization, and saturation.

**Figure 8:**
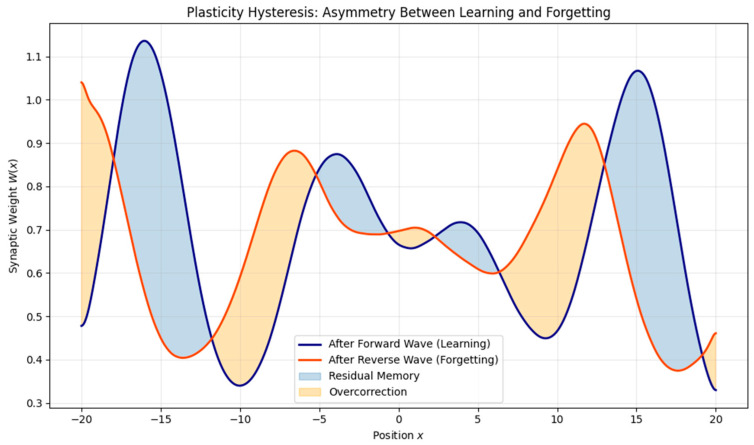
Plasticity hysteresis under forward versus reverse wave stimulation. Comparison of synaptic weights after a forward (learning) and reversed (forgetting) traveling wave. The asymmetry reveals persistent traces and overcorrection, suggesting memory consolidation and direction-sensitive synaptic adaptation.

We also examined plasticity hysteresis by reversing the wave propagation direction after learning. Results showed asymmetric changes in the weight field:

• Some memory traces persisted despite the reversal.
• Others were suppressed or inverted, indicating nonlinear memory overwrite or suppression (Figure 7 and Figure 8).

Quantification. Using the above protocol, the hysteresis loop in the $\bar{W}-I$ plane exhibited a non-zero area with ∣H∣ = 0.0389 (signed H = −0.0389) and normalized area H_norm_ = 0.3947. The reversibility index was R = 0.2396, indicating a partial recovery toward the baseline, and the trace overlap was Ω = 0.9186, showing a high but incomplete concordance between post-forward and post-reverse fields. Together, these metrics confirm path-dependent plasticity rather than the simple undoing of learning. Spatially resolved hysteresis densities H_x_ highlighted localized hot spots (Supplementary Material S2, Figures S1–S3). A small diffusion sweep (Supplementary Material S2, Table S1) further showed that increasing D_W_ reduced H_norm_ and improved R, consistent with more reversible weight dynamics.

#### 3.1.5. Functional Complexity, Robustness, and Interference

**Figure 9:**
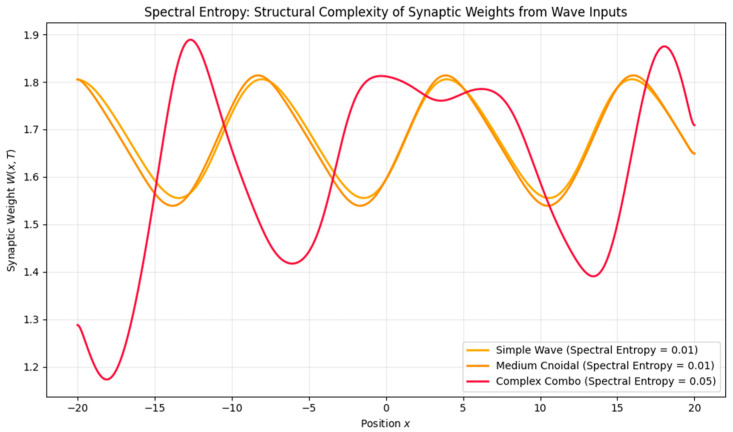
Spectral entropy of memory traces under increasing input complexity. Higher-complexity waveforms (e.g., nested or multi-scale) lead to greater spectral entropy in synaptic weights, reflecting richer, less compressible memory structures.

**Figure 10:**
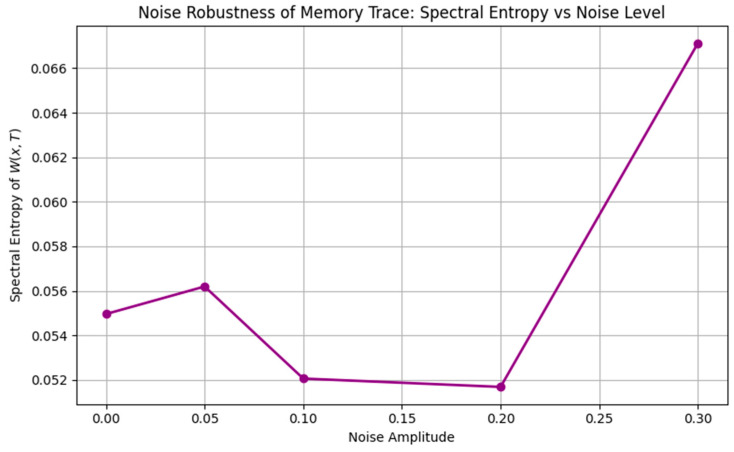
Noise robustness of synaptic plasticity patterns. Low noise preserves structure; moderate noise induces distortion; and high noise erodes memory traces entirely. This reflects biologically plausible sensitivity to cognitive load, distraction, or degradation.

**Figure 11:**
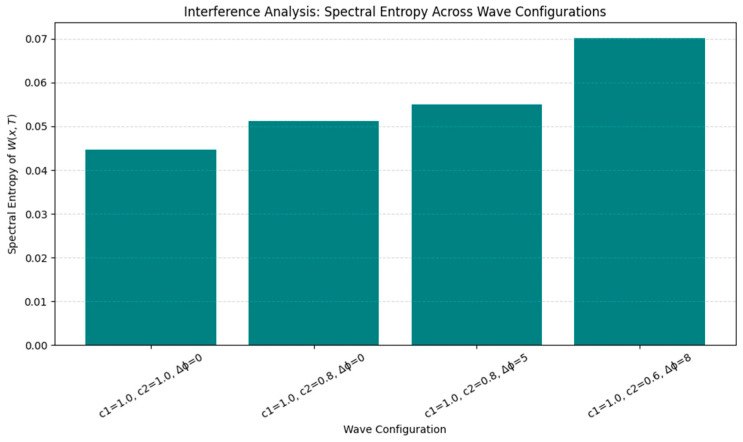
Effects of wave speed and phase mismatch on encoding fidelity. Interference from mismatched wave speeds and phase shifts increases spectral entropy and reduces spatial specificity. This models memory interference and degradation under competing or asynchronous input conditions.

To further explore the functional capacity of the plasticity system, we tested encoding under increasingly complex inputs:

• Waveform Complexity: Higher-frequency or nested inputs (e.g., theta–gamma modulation) induced higher spectral entropy, suggesting a richer, less compressible memory trace.
• Noise Robustness: Low noise preserved the memory structure, moderate noise introduced blurring, and high noise eventually erased all encoding.
• Phase and Speed Interference: Mismatched wave speeds or phase shifts elevated entropy and degraded encoding precision, modeling real-world memory interference (Figure 9, Figure 10 and Figure 11).

The simulations presented above illustrate how different waveforms and signal structures shape the evolution of synaptic plasticity over space and time. From localized solitonic inputs to complex interference patterns and noise-induced degradation, each mechanism contributes to a distinct memory encoding signature. To summarize these core behaviors and their biological analogs, Table 2 provides an overview of the principal model features, their simulated effects, and corresponding phenomena observed in neural systems.

These simulations demonstrate how nonlinear wave dynamics, when coupled to activity-dependent plasticity, can give rise to structured memory traces. We now turn to the biological interpretation of these results in the context of known neurophysiological systems.

### 3.2. Extensions and Predictions

To assess the generalizability and biological plausibility of our model, we explored three key extensions: (a) cross-frequency coupling, (b) multi-dimensional plasticity, and (c) biological fitting to real neural structures. These investigations reinforce the idea that wave-based synaptic plasticity can account for the emergence of structured, distributed memory traces seen in diverse cortical regions.

#### 3.2.1. Cross-Frequency Coupling

Oscillatory rhythms in the brain often interact across multiple timescales—for example, gamma oscillations (~30–100 Hz) modulated by slower theta rhythms (~4–8 Hz), a phenomenon implicated in hippocampal memory encoding [1,3]. To investigate how nested oscillations shape synaptic plasticity, we introduced a wave function of the form

(13) $$\psi \left(x,t\right)=[1+A_{\theta }(t)]\cdot sin\left(\omega_{\gamma }\left(x-ct\right)\right)$$

where A_θ_(t) is a theta-frequency envelope modulating a gamma carrier wave. We then compared the resulting synaptic weight profiles under three conditions: theta-only, gamma-only, and theta–gamma coupling.

**Figure 12:**
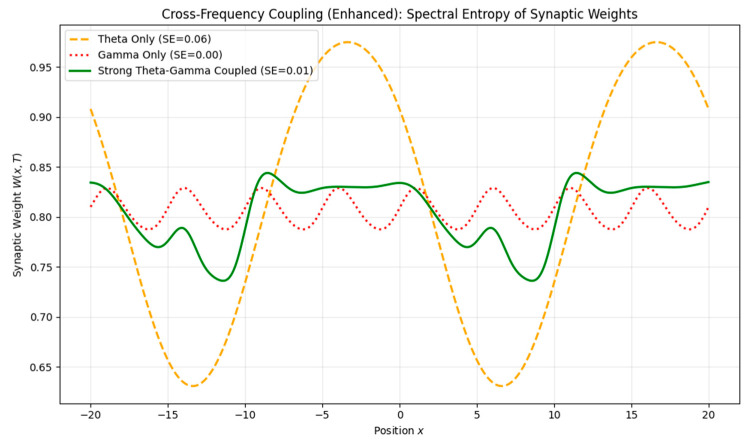
Spectral entropy of synaptic weight fields under theta-only, gamma-only, and theta–gamma coupling. Comparison of spectral entropy values for three types of rhythmic neural input. Gamma-only input produces low-complexity plasticity, while nested theta–gamma patterns induce more structured and multi-scale memory traces. This supports the role of cross-frequency coupling in organizing richer synaptic representations.

Results: Nested theta–gamma input produced synaptic weight fields with greater spatial complexity and finer granularity than either component alone. Spectral entropy increased from ~0.00 in the gamma-only case to ~0.01 under strong theta modulation, indicating an enriched multi-scale structure and supporting phase coding hypotheses in memory systems (Figure 12).

While Equation (13) implements theta–gamma nesting as the amplitude modulation, it should be regarded as a simplified proxy for hippocampal phase–amplitude coupling. In real neural recordings, PAC involves gamma bursts whose amplitudes are modulated by the theta phase. Our formulation captures the enrichment of complexity but does not yet reproduce detailed physiological PAC metrics (e.g., modulation index).

#### 3.2.2. Multi-Dimensional Plasticity

We extended the model to two spatial dimensions, simulating synaptic plasticity across a cortical sheet. Using interference between multiple traveling waves, we generated and refined the topographic maps of synaptic weights. Initial simulations with two orthogonal waves produced a grid-like plasticity structure, which was further refined by adding a third wave at 120°, forming a hexagonal tiling pattern.

**Figure 13:**
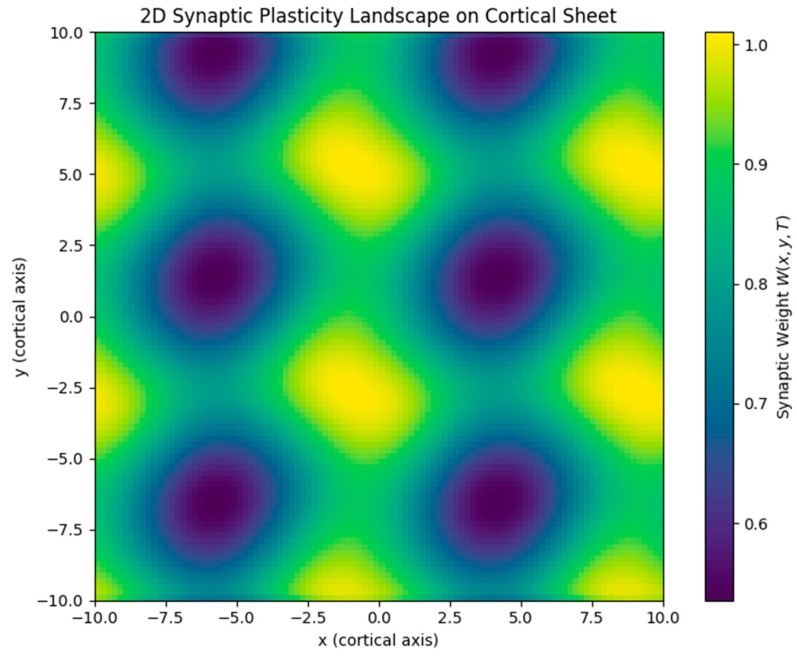
Simulated 2D synaptic weight field showing hexagonal symmetry from three-wave interference. Wave interference from three traveling waves oriented at 0°, 60°, and 120° produces periodic maxima in the plasticity field. The resulting triangular lattice structure closely resembles the hexagonal firing grid observed in medial entorhinal cortex grid cells.

Results: The resulting synaptic weight map exhibited periodic maxima arranged in a triangular lattice, closely resembling the hexagonal firing fields of grid cells in the medial entorhinal cortex [13] (Figure 13).

Hexagonal tiling arises specifically when three equal-amplitude plane waves (and their opposites) are combined at ~60° separations, producing a six-vector reciprocal lattice. This interference pattern generates triangular lattice intensity maxima, which the plasticity dynamics integrate into hexagonal weight maps. The symmetry is robust to small perturbations (±5–10°; Supplementary Material S2, Figure S4B), which preserve the approximate hexagonal spacing, but larger angular deviations (e.g., 45°/135°; Supplementary Material S2, Figure S4C) degrade the lattice and yield elongated or rectangular motifs. These results indicate that near-60° spacing is necessary and sufficient for stable grid-like structures, while tolerance to minor deviations reflects the robustness of the interference mechanism.

While these 2D results reproduce the expected hexagonal symmetry qualitatively, they have not yet been benchmarked against standard experimental metrics. In the grid cell literature, quantitative measures such as the grid score (derived from spatial autocorrelation [13]) are routinely used to validate periodicity. Similarly, for orientation maps in V1, indices such as the orientation selectivity index (OSI) are applied. Incorporating these quantitative comparisons is an essential next step to firmly establish the biological relevance of our 2D simulations.

#### 3.2.3. Biological Fitting

To validate the biological relevance of the model, we compared simulated plasticity fields with empirical data from three cortical systems:

##### Entorhinal Cortex (Grid Cells)

**Figure 14:**
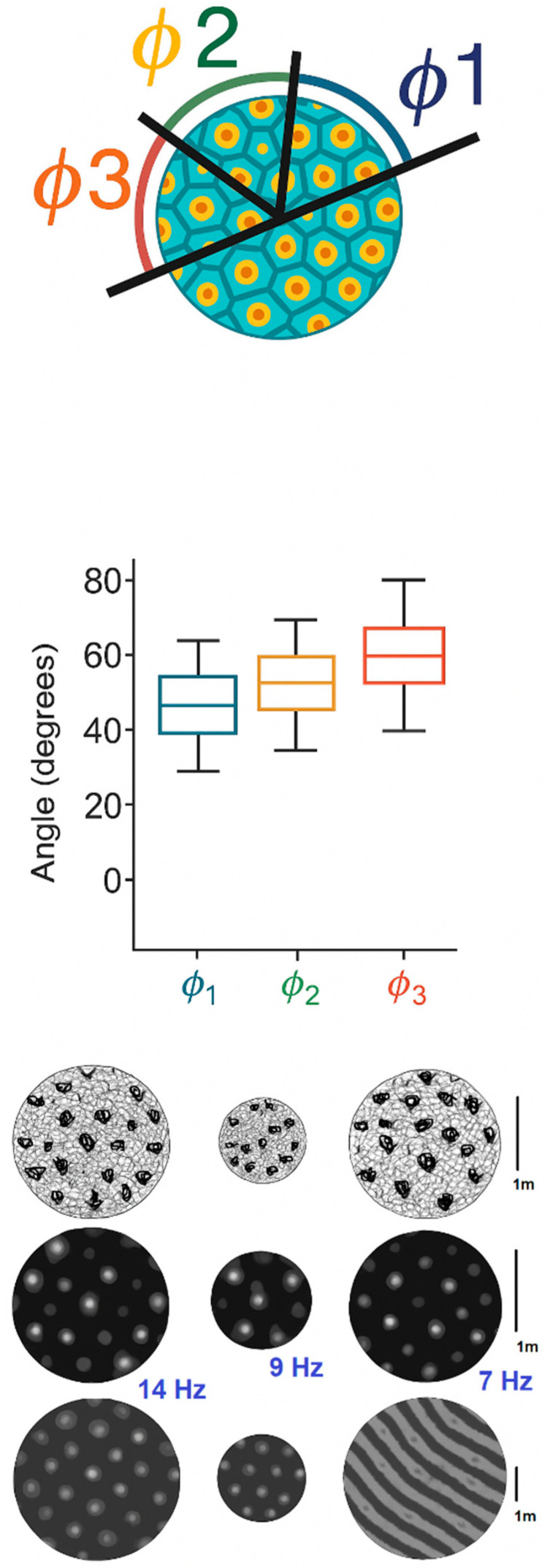
Empirical firing map of a grid cell in rodent medial entorhinal cortex (adapted from [13]). Firing rate heat map recorded during free exploration in a square arena. The pattern exhibits regular hexagonal symmetry, serving as a biological reference for the simulated interference-based plasticity shown in Figure 13.

Our hexagonal plasticity fields mirror the firing maps observed in rodents navigating 2D environments (Figure 14).

##### Hippocampus (CA1 Place Fields)

**Figure 15:**
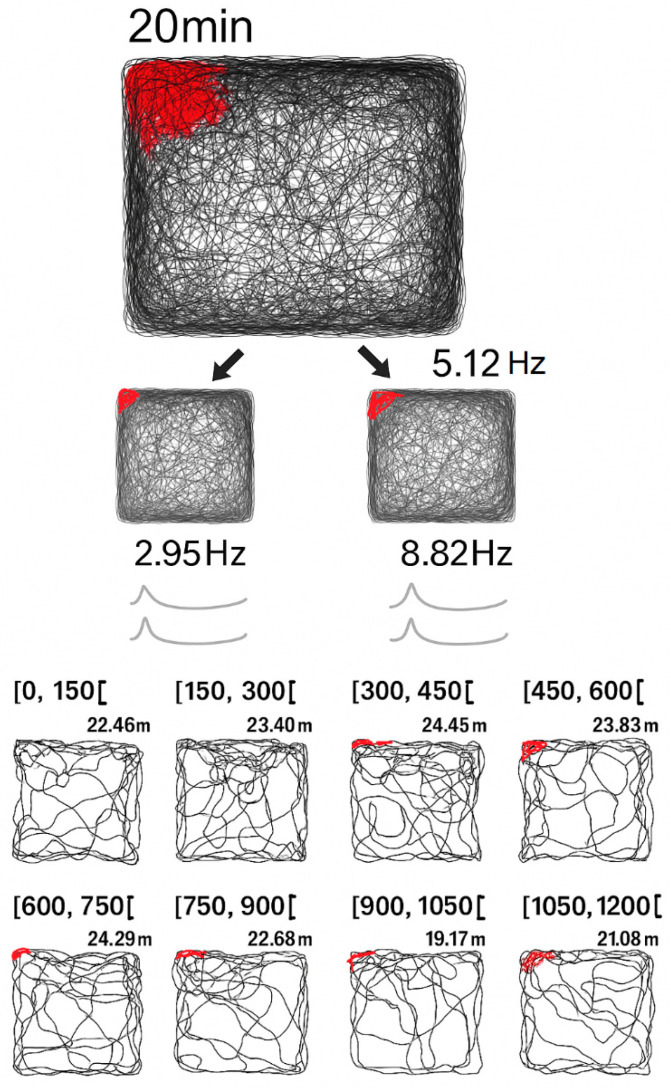
Hippocampal place field development during exploration (adapted from [14]). A rat’s foraging trajectory (black line) with superimposed spike locations (red dots) of a CA1 place cell. The field intensifies over time, reflecting gradual memory formation. This is consistent with the model’s time-dependent synaptic potentiation driven by repeated wave activation.

By modulating the wave input with Gaussian envelopes or spatial phase shifts, our model produced localized peaks in synaptic weights analogous to hippocampal place fields (Figure 15).

Red dots show spike locations, highlighting the gradual development of a place field over time. Our model reproduces a similar localized plasticity buildup under sustained, structured input:

(14) $$\frac{\partial W}{\partial t}=\rho W\left(1-\frac{W}{K}\right)-\alpha {\left|\psi \right|}^{2}W+D_{W}\Delta W$$

Repeated targeting by ∣ψ(x,t)∣^2^ induces gradual potentiation, mimicking the delayed but stable place field formation in CA1 neurons.

##### Primary Visual Cortex (V1)

The modular, pinwheel-like orientation maps in V1 emerge from self-organization during development. Our 2D interference simulations generated periodic synaptic modules analogous to V1 orientation columns.

Empirical optical imaging studies in cat primary visual cortex have revealed that iso-orientation domains are arranged in pinwheel-like patterns across the cortical surface [15]. This modular and periodic organization parallels the simulated plasticity patterns generated through 2D wave interference in our model.

Summary: These results indicate that wave-driven synaptic plasticity, when extended to two dimensions and enriched with biologically relevant features (like cross-frequency coupling), can replicate both the structural and functional motifs observed in the brain. The model provides a mechanistic bridge between nonlinear physical dynamics and neural map self-organization, with implications for memory, navigation, and sensory processing.

Taken together, these extensions reinforce the versatility of the model in replicating diverse neural structures and dynamics. We now reflect on the broader theoretical implications, limitations, and future directions for this line of work.

To consolidate the core findings of our simulations and their biological relevance, Table 3 summarizes the key features of the model, the corresponding plasticity outcomes, and their analogs in known neural systems.

## 4. Discussion

### 4.1. Biological Implications

The patterns observed in our simulations resonate with several well-documented neural phenomena. In this section, we interpret these results in the context of memory formation, cortical organization, and biologically realistic learning dynamics.

The results of our model offer several biologically grounded insights into how structured neural activity can give rise to long-term memory patterns through wave-driven synaptic plasticity.

First, the model suggests a plausible mechanism for rhythm-driven memory formation, aligning with empirical observations of theta (4–8 Hz) and gamma (30–100 Hz) oscillations in the hippocampus and cortex. These oscillatory dynamics are known to play a key role in organizing the timing of synaptic updates and guiding the flow of information during learning and recall [1,2].

Importantly, the model makes explicit predictions regarding the amplitude and phase components of neural activity. The amplitude ∣ψ∣ maps onto population-level activity strength, suggesting that regions of high synaptic potentiation should coincide with elevated, multiunit firing rates or LFP power. The phase of ψ represents the temporal alignment of oscillations, implying that wave interference and phase resetting in the model could be observed experimentally as changes in the phase locking value (PLV) or cross-frequency coupling. Thus, the framework predicts that spatially structured memory traces should co-occur with measurable phase–amplitude signatures in hippocampal and cortical recordings, providing a direct avenue for empirical testing.

Second, the emergence of spatially periodic synaptic patterns, particularly under cnoidal wave input or 2D interference, mirrors the structured organization observed in biological systems. Notably, this includes grid cell firing patterns in the entorhinal cortex [3], orientation maps in the primary visual cortex (V1) [4], and modular synaptic architectures in cortical columns.

Third, our approach provides a physics-based explanation for how traveling wave propagation in neural tissue can induce long-term changes in synaptic efficacy. Unlike models that rely on pointwise spike correlations, this framework emphasizes the spatiotemporal structure of input signals, allowing distributed memory traces to form naturally as emergent patterns of the activit–plasticity interaction.

Furthermore, the model highlights the role of interference between overlapping waveforms as a potential mechanism for memory competition, contextual distortion, and forgetting under noisy conditions. This aligns with behavioral and electrophysiological studies showing that overlapping experiences can degrade memory specificity or trigger retroactive interference [5].

Our quantitative analyses further bridge mathematical output with cognitive function: higher entropy corresponds to increased memory complexity; correlation with neural activity tracks fidelity; and spectral content maps to encoding granularity. These metrics provide interpretable, biologically relevant markers of the learning state and memory structure.

The time-resolved dynamics of synaptic weights show a gradual buildup of structure in response to sustained input followed by stabilization—mirroring empirically observed stages of memory encoding and consolidation [11]. Crucially, the system does not exhibit runaway plasticity or saturation, which supports its plausibility as a stable learning substrate.

Importantly, the model also reveals plasticity hysteresis, wherein the reversal of the input direction (e.g., during “forgetting”) does not simply undo prior learning. Instead, synaptic weights show asymmetric adaptation: residual potentiation may persist, while certain regions undergo overcorrection or suppression. This dynamic is consistent with the asymmetric learning and reconsolidation processes found in studies of extinction, memory updating, and interference [12].

Finally, the model’s sensitivity to input complexity and noise levels reveals a form of functional selectivity: the system preferentially encodes richly structured, persistent inputs while filtering out redundant or noisy patterns. This supports the idea of information compression and prioritization during memory formation, and parallels findings in both computational and biological models of synaptic plasticity [13].

The biological plausibility of these results invites further exploration into more complex and realistic scenarios, including hierarchical oscillations, multi-dimensional structures, and alignment with empirical data—all of which are addressed in the following section.

### 4.2. General Analysis

In this work, we introduced a theoretical framework that models synaptic plasticity as the product of nonlinear wave propagation in a fractal space-time environment, derived from the Scale Relativity Theory (SRT). By coupling Schrödinger-type neural activity equations to a reaction–diffusion plasticity model, we demonstrated how structured, dynamic memory traces can emerge from spatiotemporal patterns of neural activation.

#### 4.2.1. Summary and Interpretation of Key Findings

Our primary finding is that different waveforms encode distinct types of synaptic structures:

• Soliton-like waves induce localized, persistent plasticity, aligning with the observations of sharp, spatially confined representations such as hippocampal place fields [14].
• Cnoidal (periodic) waves, by contrast, produce regularly spaced plasticity landscapes resembling grid cell patterns in the entorhinal cortex [13].

When multiple waveforms interact—with or without noise—interference patterns emerge that shape complex, non-uniform synaptic fields. These patterns model how the brain might integrate competing or overlapping experiences or form composite memory representations from multiple input streams. Quantitative measures such as entropy, cross-correlation, and spectral analysis confirmed that the resulting memory traces are structured, resilient to low noise, and sensitive to wave geometry and input complexity.

We also found that memory formation in this model is gradual, self-regulating, and saturates without divergence, supporting its biological plausibility. The presence of hysteresis in synaptic updating further suggests that forgetting or memory modification is path-dependent rather than a simple reversal of learning—a phenomenon well-documented in reconsolidation and extinction studies [17,18].

#### 4.2.2. Relationship with Existing Work

Our approach bridges two major themes in neuroscience:

• Neural waves and rhythms in information flow. Empirical studies have shown that traveling cortical waves [19,20] and nested oscillations such as theta–gamma coupling [11,21] coordinate neural activity across spatial and temporal scales and are crucial for learning, working memory, and attention [22]. By modeling waves as carriers of both energy and structure, our work provides a formal mechanism for how such dynamics might imprint memory onto synaptic substrates.
• Plasticity mechanisms and memory models. Classical accounts such as spike-timing-dependent plasticity (STDP) [3,4], cascade models of synaptic memory [23], and homeostatic plasticity frameworks [24] explain how local timing rules, probabilistic cascades, or stability constraints shape synaptic strength. Our reaction–diffusion rule, coupled with nonlinear wave dynamics, complements these frameworks by emphasizing the spatially distributed aspects of plasticity. Unlike pointwise or pair-based rules, it explicitly incorporates diffusion and wave interference, thereby generating map-like synaptic organizations and interference-driven distortions.

In doing so, our SRT-based model does not aim to replace conventional STDP or attractor-like models but rather to extend them by highlighting how fractal and wave-based propagation may provide an additional organizing principle. This positioning is especially important given that the assumption of fractal geodesics remains speculative and requires empirical testing (see Section 4.2.3). In this way, we address a gap between mechanistic biophysical models and systems-level functional theories of learning and memory [25].

We also note that other physics-inspired frameworks have been proposed that bridge neuroscience and theoretical physics, including quantum mechanics, QFT, and lattice field theory approaches [7,8]. These models, which often describe neural fields as quantum-like systems or effective field theories, provide valuable alternative perspectives. Our contribution is complementary: rather than importing field-theoretic formalisms wholesale, we leverage the geometric framework of the SRT to propose fractal geodesics as a candidate organizing principle for wave-driven plasticity.

In addition, fractal signatures in neural structures and dynamics have long been documented [9,10], and our framework should be understood as an extension of the literature that embeds such fractality within the SRT formalism.

#### 4.2.3. Limitations and Assumptions

Several limitations of the current model should be acknowledged:

1. Dimensionality. Most simulations were conducted in 1D or simplified 2D environments. While these are sufficient for modeling linear cortical strips or hippocampal slices, full cortical modeling would require extending this to 3D networks with heterogeneous geometries. A further limitation is that our 2D results have thus far been evaluated only qualitatively. Although the simulated maps reproduce hexagonal grid-like tiling and modular V1-like domains, they have not been quantitatively benchmarked against biological data. Established metrics such as the grid score (from spatial autocorrelation analyses) and the orientation selectivity index (OSI) would provide stronger validation and enable parameter fitting across datasets. We regard this as a key direction for future work. Furthermore, our analysis shows that the hexagonal symmetry observed in 2D simulations is contingent on the relative orientation of the component waves. While the grid pattern is robust to small angular perturbations (±5–10°), larger deviations disrupt the symmetry. This suggests that interference-driven map formation depends on geometric constraints that may reflect or limit the flexibility of biological grid cell formation mechanisms.
2. Neuronal specificity. The model operates on neural field amplitudes, abstracting away from individual neuron types, dendritic dynamics, or neurotransmitter effects. It lacks the granularity of models like Izhikevich spiking networks [26] or voltage-based STDP [24].
3. Simplified plasticity. Although the logistic reaction–diffusion model captures important qualitative dynamics, it does not account for complex biological processes such as neuromodulatory gating (e.g., dopamine, acetylcholine), synaptic tagging, or glial influence. The framework should therefore be regarded as a minimal proof-of-concept rather than a complete biophysical description of synaptic plasticity.
4. No direct behavioral link. Our simulations do not yet connect memory traces to behavior or decision-making. Future work should investigate how structured plasticity fields relate to navigation, recall, or pattern recognition.
5. Speculative assumption of fractal geodesics. A core feature of the model is the assumption that neural signal propagation can be described by fractal geodesics in non-differentiable space-time, as formalized in the Scale Relativity Theory [1,5]. While fractal and scale-free properties are widely observed in neural anatomy and dynamics, there is no direct evidence from neurophysiological recordings that signals follow such geodesic trajectories. This assumption should therefore be framed as speculative and hypothesis-generating.
6. Empirical comparison and validation. Although our qualitative comparisons show structural parallels with grid cells [13], CA1 place fields [14], and V1 orientation maps [15], these remain preliminary. Stronger validation will require quantitative fitting across datasets and experimental tests of the model’s predictions.
7. Robustness and universality. The present results demonstrate a tolerance to noise and the repeated emergence of structured motifs, but these behaviors are simulation-based and parameter-dependent. Claims of robustness and universality should therefore be moderated until supported by systematic sensitivity analyses.
8. Benchmarking against established models. The framework was not explicitly compared with conventional accounts such as oscillatory and traveling wave models [3,4], short-term dynamics and STDP [16], or engram-level studies linking plasticity to memory [18]. Future benchmarking against these well-established frameworks will be critical to clarify what is unique about the SRT formulation and where it aligns with or diverges from existing theories.
9. Hysteresis quantification. Although we have now introduced formal hysteresis metrics—loop area ∣H∣, normalized hysteresis index H_norm_, reversibility index R, and trace overlap Ω—these are based on simulations. Validation against experimental data on synaptic reversibility (e.g., extinction and reconsolidation studies [12,13]) will be essential to establish biological relevance.
10. Cross-frequency coupling. Our implementation of the theta–gamma interaction (Equation (13)) represents a minimal amplitude-modulated wave. This does not fully reflect the phase–amplitude coupling observed in hippocampal circuits, where gamma bursts are locked to specific theta phases. Future extensions should incorporate more physiologically grounded PAC models (e.g., modulation index-based parametrizations) to better connect with experimental data.

#### 4.2.4. Future Directions

Building on this foundation, several extensions are possible:

• Cross-frequency coupling: Preliminary results with nested theta–gamma waves suggest rich possibilities for encoding sequences or hierarchical memory structures. This should be systematically explored using empirical oscillatory parameters [21].
• Integration with spiking models: Coupling the wave plasticity model to spiking networks could offer a bridge between abstract field dynamics and biologically detailed simulations [26].
• Realistic cortical topologies: Mapping our interference-based plasticity model onto anatomically accurate cortical surfaces could explain the map formation in V1, auditory tonotopy, or motor coordination patterns.
• Behavioral modeling: Memory-driven behavior, including path planning, goal-directed recall, or contextual modulation, could be simulated by feeding wave-shaped plasticity fields into higher-order decision models.
• Validation through imaging and optogenetics: Experimental designs could use patterned stimulation to induce traveling waves and observe whether similar plasticity patterns emerge in vivo [20].
• Experimental validation. Although our work is simulation-based, it generates several testable predictions that can guide empirical studies. First, targeted optogenetic stimulation patterns could be used to induce traveling or interfering waves in cortical tissue; our model predicts that such patterned inputs would leave structured synaptic weight maps resembling grid-like or modular arrangements. Second, in vivo hippocampal recordings under theta–gamma coupling paradigms could be analyzed for plasticity hysteresis, with partial reversibility expected during extinction or reversal learning. Third, quantitative analyses of cortical maps (grid scores, orientation selectivity indices) under controlled perturbations of oscillatory phase relationships could directly assess whether interference geometry governs the emergence of spatial symmetries, as predicted here. These paradigms provide concrete routes to falsify or refine the framework and to bridge theory with experiment. To facilitate empirical testing, we summarize, in Supplementary Material S2, Table S2, a set of model predictions alongside concrete experimental paradigms. These include the optogenetic induction of traveling waves, grid cell stimulation under interference angles, closed-loop theta–gamma modulation, and extinction/reversal protocols for probing hysteresis. This table provides a roadmap for the validation and falsification of the present framework.

#### 4.2.5. Implications for Neuroscience and AI

The framework introduced here offers a compelling link between nonlinear dynamics, geometric structures, and information encoding. It provides a foundation for new models in cognitive neuroscience, where memory is not merely stored but dynamically sculpted by the structure of neural activity. Beyond neuroscience, the model has implications for machine learning and neuromorphic engineering, where principles such as wave interference, fractal modulation, and distributed plasticity could inform the development of robust, self-organizing computational architectures.

This approach contributes to the field by

• Introducing a novel mathematical formalism for synaptic plasticity grounded in the Scale Relativity Theory.
• Demonstrating explanatory power across multiple scales, from synaptic-level learning to map-like cortical organization.
• Offering testable predictions and clear paths for extension through both computational refinement and empirical validation.

## 5. Conclusions

This work presents a novel theoretical framework that links neural wave dynamics with synaptic plasticity, grounded in the principles of the Scale Relativity Theory (SRT). By modeling neural activity as propagating along fractal geodesics, we derived nonlinear wave equations whose solutions—such as solitons and cnoidal waves—interact with a biologically plausible plasticity mechanism to induce structured, spatially patterned memory traces.

Through simulations, we demonstrated that

• Localized waveforms generate sharp, discrete potentiation zones, modeling features like place fields;
• Periodic waves yield grid-like or modular synaptic architectures, paralleling grid cells and cortical maps;
• Wave interference and noise produce complex plasticity landscapes, offering a mechanism for memory interference, competition, and contextual encoding.

Quantitative analyses of the resulting synaptic fields revealed a balance between informational richness and structural stability, as well as resilience to low levels of noise—traits essential for reliable memory formation.

Importantly, the model captures key biological phenomena:

• Time-dependent plasticity buildup and saturation;
• Asymmetric forgetting and hysteresis;
• Selectivity for structured input over unstructured or transient signals.

These findings suggest that wave-driven plasticity in a fractal substrate may serve as a general mechanism for rhythm-organized, spatially distributed learning in the brain. This approach not only provides a physically grounded explanation for observed memory patterns but also opens new directions for

• Experimental validation using patterned stimulation and imaging;
• Computational extension to more realistic, high-dimensional neural systems;
• Application to neuromorphic computing where robust, distributed memory is a central design challenge.

Altogether, this framework contributes a unifying theoretical model that connects geometry, dynamics, and plasticity in service of understanding how structured memory can emerge from complex neural activity.

## Figures and Tables

**Table 1:** Mapping of model components to biological counterparts. Each element of the proposed wave–plasticity framework corresponds to a biologically plausible mechanism or observed neural phenomenon. The neural activity field ψ(x,t) is interpreted as a population-level excitation signal, while ∣ψ∣^2^ modulates synaptic plasticity through a reaction–diffusion mechanism. Traveling and periodic wave solutions align with known spatial and rhythmic structures in the brain, such as place fields and grid cells. This table serves as a conceptual bridge between the mathematical formalism and the neurobiological processes it aims to model.

Model Component	Biological Interpretation	Known Phenomenon Modeled
Soliton wave (localized ψ)	Burst of localized neural activity (e.g., sharp-wave ripple)	CA1 place fields
Cnoidal wave (periodic ψ)	Oscillatory input/spatial periodicity	Grid cell firing in mEC
ψ(x, t): Neural activity field	Distributed excitation density across space-time	Population-level neural field/LFP analog
∣ψ∣^2^: Activity intensity	Proxy for metabolic/neural drive driving plasticity	Hebbian modulation by activity
Reaction–diffusion in W(x, t)	Synaptic strength modulated by activity + lateral spread	Cortical map self-organization
Interference patterns	Competing inputs or memories interacting	Memory interference and consolidation
DW (plasticity diffusion)	Lateral spread of synaptic effects	Dendritic/synaptic propagation of plastic change

**Table 2:** Summary of core model behaviors and their biological relevance. This table summarizes the core results obtained from the model in one-dimensional simulations, linking each wave-driven mechanism to observed neural encoding phenomena in hippocampal and cortical systems.

Model Feature	Simulated Outcome	Biological Correspondence
Soliton wave input	Localized, persistent synaptic potentiation	Hippocampal place fields (CA1)
Cnoidal wave input	Periodic synaptic weight landscape	Grid cell firing structure (entorhinal cortex)
Wave interference (cnoidal × 2)	Constructive/destructive plasticity modulation	Memory competition and interference effects
Time-dependent wave input	Gradual potentiation; saturation without divergence	Temporal dynamics of learning and consolidation
Wave reversal	Asymmetric forgetting; residual traces and overcorrection	Hysteresis and reconsolidation in synaptic memory
Noise scaling	Degradation of structure with high noise	Cognitive load, distractor interference, and forgetting
Input complexity variation	Increase in spectral entropy with waveform complexity	Information richness and selective memory encoding
Phase mismatch/wave speed shift	Reduced encoding fidelity and periodic distortion	Memory degradation under asynchronous input

**Table 3:** Summary of extended model scenarios and their alignment with empirical brain data. This table highlights results from advanced simulations in two dimensions and cross-frequency conditions. The plasticity patterns produced by these mechanisms closely resemble known spatial and functional maps in the hippocampus, entorhinal cortex, and primary visual cortex.

Extended Model Feature	Simulated Outcome	Biological Correspondence
Theta–gamma nested input	High spectral entropy; multi-scale trace complexity	Cross-frequency coupling in hippocampal memory encoding
2D wave interference (3 angles)	Hexagonal grid of synaptic weights	Grid cell lattice pattern in medial entorhinal cortex
Gaussian-modulated wave input	Localized potentiation zones; late buildup	CA1 place field development
Multi-wave + noise interaction	Irregular, interference-driven plasticity structure	Memory overlap and competitive encoding under noise
Periodic 2D maps	Modular plasticity zones; columnar tiling	V1 orientation maps and pinwheel structures

## Data Availability

No new data were created or analyzed in this study. Data sharing is not applicable to this article.

## Supplementary Materials

The following supporting information can be downloaded at: https://www.mdpi.com/article/10.3390/brainsci15101037/s1, Supplementary Material S1: Derivation of Equation (4) from the SRT covariant derivative. Supplementary Material S2: Figures and Tables.
