# Supplementary material for: Fractal Neural Dynamics and Memory Encoding Through Scale Relativity
Source: Brain Sci. 2025 Sep 24;15(10):1037. doi: 10.3390/brainsci15101037 (PMC12563330; doi:10.3390/brainsci15101037)
Supplement: Supplementary file 1 [file brainsci-15-01037-s001.zip › S1.pdf]

Supplementary Material S1. Derivation of Equation (4) from the SRT covariant derivative

S1.1 Covariant derivative in Scale Relativity.

In the Scale Relativity Theory (SRT), the dynamics of a signal carrier along a non-differentiable fractal trajectory are described using the complex covariant derivative:

$$\frac{\hat{d}}{dt} = \left(\frac{\partial}{\partial t} + \boldsymbol{V} \cdot \nabla - iD\Delta\right) \tag{3}$$

where D > 0 is a diffusion-like coefficient and $\boldsymbol{V} = \boldsymbol{v} - i\,\boldsymbol{u}$ is the complex velocity.

S1.2 Hydrodynamic substitution.

Following Nottale (1993) [5], we introduce a complex wavefunction

$$\psi = \sqrt{\rho} e^{iS/(2D)}$$

with $\rho = |\psi|^2$ representing an activity density and S a phase potential. This leads to

$$\boldsymbol{V} = 2D\nabla(\ln\psi)$$

S1.3 Geodesic equation.

The SRT geodesic equation is

$$\frac{\hat{d}}{dt}\boldsymbol{V} = -\nabla\Phi \tag{3a}$$

where Φ(x,t) is an external potential (e.g., external drive or medium heterogeneity).

S1.4 Complex Hamilton–Jacobi form.

Integrating the velocity field yields a complex action S, leading to

$$\partial_t S + \frac{1}{2}(\nabla S)^2 - iD\Delta S + \Phi + G(\rho) = 0$$

where $G(\rho)$ denotes possible nonlinear self-interaction terms.

S1.5 Continuity and momentum balance.

Separating real and imaginary parts gives

- Continuity equation:

$$\partial_t \rho + \nabla \cdot (\rho \boldsymbol{v}) = 0$$

- Momentum equation with an additional "quantum-like" potential:

$$\partial_t \boldsymbol{v} + (\boldsymbol{v} \cdot \boldsymbol{\nabla})\boldsymbol{v} = -\nabla\Phi - \nabla \mathrm{Q}(\rho)$$

with $\mathrm{Q}(\rho) = -2D^2 \frac{\Delta\sqrt{\rho}}{\sqrt{\rho}}$.

S1.6 Recombination into Schrödinger-type equation.

Combining the continuity and momentum terms into the complex field ψ yields the nonlinear Schrödinger-type equation:

$$i2D\partial_t\psi = -2D^2\Delta\psi + \Phi\psi + g|\psi|^2\psi \tag{4}$$

where g parameterizes the nonlinear self-interaction $G(\rho)$.

S1.7 Notes and assumptions.

- Assumes irrotational velocity ($\nabla \times \mathbf{V} = 0$).
- Boundary conditions: Vanishing surface terms at infinity or periodic domains.
- For $g = 0$, Eq. (4) reduces to the linear Schrödinger equation.
- Biological interpretation: $|\psi|$ is the activity amplitude (population-rate proxy), arg ψ is the oscillatory phase, and Φ encodes the structured input.
