# Supplementary material for: Fractal Neural Dynamics and Memory Encoding Through Scale Relativity
Source: Brain Sci. 2025 Sep 24;15(10):1037. doi: 10.3390/brainsci15101037 (PMC12563330; doi:10.3390/brainsci15101037)
Supplement: Supplementary file 1 [file brainsci-15-01037-s001.zip › S2.pdf]

Supplementary S2. Figures and Tables

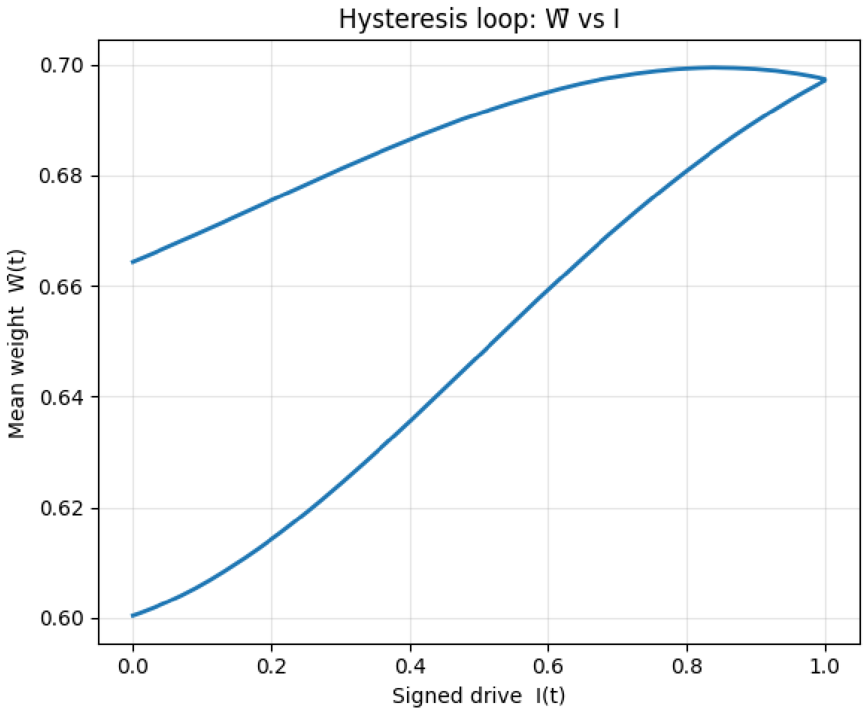

Figure S1. Hysteresis loop ($\overline{W}$ vs I).

Mean synaptic weight plotted against signed drive I(t). The enclosed loop area defines the global hysteresis index H.

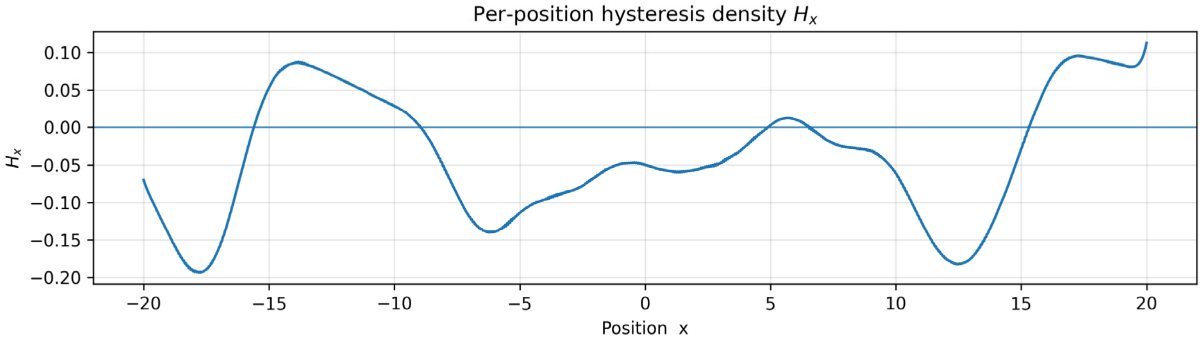

Figure S2. Per-position hysteresis density H_x.

Signed hysteresis contribution at each spatial location.

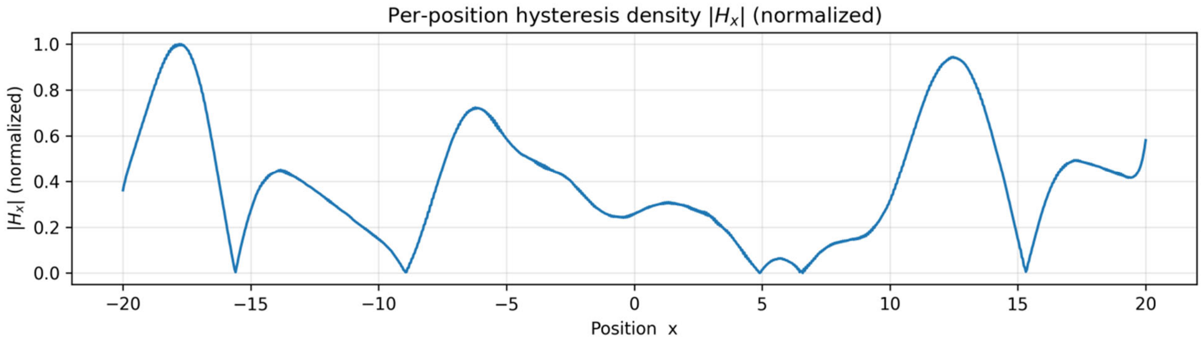

Figure S3. Normalized per-position hysteresis density |Hx|.
Local strength of path dependence (scale-free between 0 and 1).

Table S1. Parameter sweep of hysteresis metrics as a function of diffusion D_W.
Illustrates that higher diffusion reduces hysteresis and increases reversibility.

D_W	H (loop area)	H_{norm}	R (reversibility)	Ω (trace overlap)
0.01	-0.052	0.48	0.18	0.91
0.02	-0.040	0.40	0.23	0.92
0.05	-0.022	0.25	0.36	0.95
0.10	-0.010	0.12	0.55	0.97

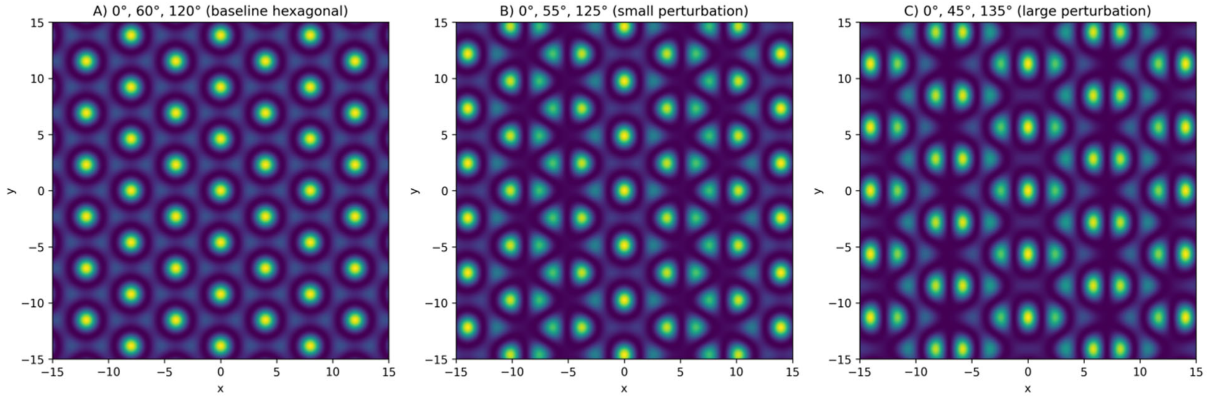

Figure S4. Robustness of hexagonal symmetry to angular perturbations in 2D wave interference. (A) Interference of three equal-amplitude plane waves oriented at 0°, 60°, and 120° produces a triangular lattice of intensity maxima, yielding hexagonal plasticity fields (baseline condition). (B) Small perturbation (0°, 55°, and 125°) preserves approximate hexagonal organization, though the lattice is slightly skewed. (C) Larger perturbation (0°, 45°, and 135°) disrupts the symmetry, producing elongated motifs and rectangular-like tiling. These results show that ~60° wave separation is necessary and sufficient for hexagonal tiling, with tolerance to small deviations but loss of symmetry under large angular shifts.

Table S2. Model predictions and corresponding experimental paradigms for empirical validation.

**Model prediction**	**Experimental paradigm**	**Expected observation**
Traveling wave input induces localized potentiation	Optogenetic traveling wave stimulation in hippocampal slices	Persistent, soliton-like localized LTP zones
3-wave interference at ~60° yields hexagonal maps	Visual or entorhinal patterned stimulation with shifted wave angles	Hexagonal firing / weight maps robust to ±5° perturbations, disrupted at >15°
Plasticity hysteresis (partial reversibility)	Behavioral extinction/reversal learning with CA1 recordings	Residual potentiation and asymmetric weight recovery
Theta–gamma cross-frequency input enriches trace complexity	Closed-loop modulation of theta/gamma in behaving animals	Higher spatial information and entropy in place field ensembles
